# Subepithelial Corneal Deposits Associated with Exemestane

**DOI:** 10.1155/2020/5703164

**Published:** 2020-06-26

**Authors:** Annahita Amireskandari, Elena Nguyen, David Hinkle, Thomas Mauger

**Affiliations:** Department of Ophthalmology, West Virginia University, USA

## Abstract

This is a case report of corneal deposits noted in a 69-year-old female patient taking the aromatase inhibitor, exemestane, after undergoing a mastectomy and chemotherapy for breast cancer. The patient presented to our eye clinic for a new-onset floater in one eye, and bilateral subepithelial opacities were found incidentally on exam. The patient completed a 5-year course of the medication shortly after her initial visit with us and was noted to have a slight improvement in the density of the opacities on a follow-up visit 3 months later. We believe these corneal changes were most likely secondary to exemestane. The effect of aromatase inhibitors on the eye deserves further exploration as an increasing number of patients are prescribed these medications.

## 1. Introduction

Exemestane (trade name Aromasin (Pfizer)) is an irreversible aromatase inhibitor (AI) used as an adjuvant treatment for postmenopausal women with estrogen receptor-positive breast cancer [1–3]. Several studies have reported on the ocular side effects of aromatase inhibitors, with the most common being ocular surface disease [4–6]. A case report by Papathanassiou et al. [5] reported corneal epithelial deposits associated with exemestane. Here, we discuss a patient on exemestane with development of subepithelial corneal opacities.

## 2. Case Report

A 69-year-old female presented to the ophthalmology clinic with a new onset of a floater in her right eye. Her medical history was notable for stage 1 estrogen, progesterone, and HER2/neu-positive invasive ductal carcinoma of the left breast diagnosed 5 years prior. She had completed 12 weeks of adjuvant chemotherapy with Taxol (paclitaxel, Bristol-Myers Squibb) and Herceptin (trastuzumab, Roche) and was in her fifth and final year of Aromasin (exemestane) when she presented to us. The only other medication she was taking at the time of presentation to our clinic was bisoprolol-hydrochlorothiazide for hypertension, which was well controlled. She had no other significant systemic illnesses. Additionally, she denied a personal or family history of eye disease.

**Figure 1:**
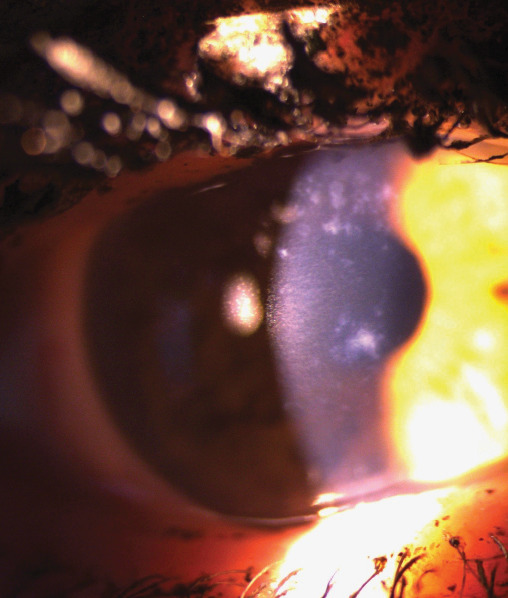
Slit lamp photograph of the right cornea.

**Figure 2:**
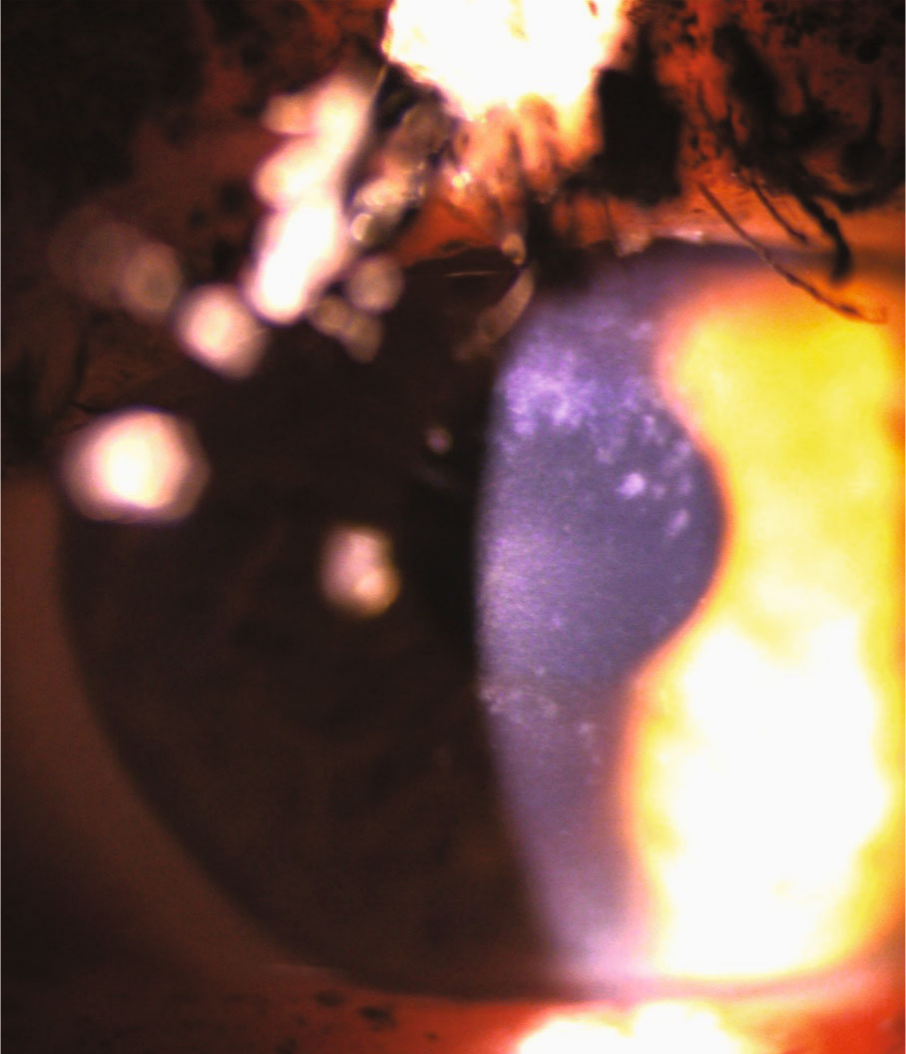
Slit lamp photograph of the left cornea.

At her initial visit to the retina clinic, she was noted to have uncorrected Snellen distance visual acuities of 20/30-2 OD and 20/25-2 OS. Her lids, adnexa, and conjunctiva were all normal. She was noted to have bilateral diffuse subepithelial corneal opacities. The deposits ranged in size from pinpoint to 0.5 mm with poorly demarcated edges. They were hazy and whitish in color, varying in density, and best visualized with a narrow slit beam. The corneal stroma and endothelium appeared normal. There was no staining with fluorescein dye. Schirmer's testing with anesthetic was 8 mm on the right and 5 mm on the left. Corneal sensation was intact bilaterally. On dilated fundus exam, she was noted to have inferotemporal retinoschisis bilaterally. The decision was made to observe the retinoschisis, and she was referred to the cornea service for further evaluation. It was thought that the subepithelial corneal irregularities were most likely secondary to exemestane. She completed 5 years of exemestane the following month, was seen at a follow-up visit, and was noted to have a posterior vitreous detachment in the right eye. The corneal findings were stable. She was seen again 3 months later after stopping the exemestane, and the subepithelial opacities were noted to have a more annular pattern and were less dense compared to prior visits. Slit lamp photographs of the right (Figure 1) and left eyes (Figure 2) were taken at this visit.

## 3. Discussion

The corneal abnormalities noted in this patient were found incidentally when she presented with a new-onset floater. We were unable to obtain any ophthalmologic records from the patient's previous eye care providers so the duration of her clinical findings remains unknown. Given the use of exemestane at the time of diagnosis and improvement after cessation of the medication, we believe this was the most likely cause of the subepithelial opacities noted on her exam.

Exemestane is an irreversible inhibitor of aromatase, the enzyme responsible for the conversion of androgens to estrogens. Although estrogen receptors are more readily associated with reproductive organs, they are also known to be present in the cornea, lens, retina, meibomian glands, and the lacrimal glands [7–11]. Several studies have reported on adverse ocular effects of AIs [12–14]. Inglis et al. [4] used the Ocular Surface Disease Index questionnaire and found a significantly higher rate of self-reported dry eye disease in patients taking AIs compared to controls. Gibson et al. noted no significant difference in ocular surface symptoms in AI users compared to controls but did note worse meibomian gland expressibility and pain perception in AI users [13]. Others have also reported increased rates of anterior segment pathology including meibomian gland dysfunction [14], blepharitis, superficial punctate keratitis, conjunctival injection [6, 14], and Sjögren's syndrome in AI users [15]. One isolated case report documented epithelial deposits associated with exemestane use [5]. These deposits were described as intraepithelial microcysts, while those of our patient appeared to be subepithelial opacities. Papathanassiou et al. [5] hypothesized that the corneal changes noted in their patient were a sign of impaired epithelium due to an inhibitory effect of exemestane on limbal stem cells, citing research that estrogens enhance stem cell survival and proliferation [16].

Eisner et al. [17] examined the effects of anastrazole, another commonly used AI, on the posterior segment. They observed an increased rate of retinal hemorrhages and vitreoretinal traction in patients taking anastrazole compared to patients taking tamoxifen and controls [17]. They hypothesized that the hemorrhages were a result of traction, and the higher rate of traction was likely due to accelerated aging from estrogen depletion, noting that posterior vitreous detachments and macular holes are more common in postmenopausal women than men [18]. Epstein also reported a case of a macular hole associated with the initiation of exemestane [19]. Interestingly, our patient initially presented with a new floater, was noted to have retinoschisis, and was subsequently found to have a posterior vitreous detachment. Though there are no published reports of retinoschisis and AI use, it is difficult to assess whether either of these posterior segment changes was the result of normal aging or if this process was accelerated by a reduction in circulating estrogen.

The exact role of estrogen on the eye is still being investigated. Though many studies attribute the adverse effects of AIs to low estrogen levels, the mechanism is likely more complex and related to the balance between estrogens, androgens, and other circulating hormones [10, 20–22].

It is also worth discussing Taxol (paclitaxel) and Herceptin (trastuzumab) as our patient had previously been treated with these agents. Given that these medications had been completed more than 4 years prior to her presentation, we believe that they were unlikely to be the cause of her corneal findings. And, while retinal and optic nerve toxicities are known adverse effects of paclitaxel, we are not aware of corneal toxicity as a reported side effect [23–27]. Herceptin and related drugs, however, have been associated with adverse effects on the anterior segment with dry eye, epiphora, blurred vision, and conjunctivitis being the most common [12, 28, 29]. It is not surprising that these drugs might have an effect on the ocular surface given the expression of HER receptors by the corneal, limbal, and conjunctival epithelia [30] and the role of HER family tyrosine kinase receptors on cell differentiation, proliferation, and migration [31, 32].

Tamoxifen, a selective estrogen receptor modulator which is commonly given prior to initiation of exemestane, has been associated with corneal deposits [33, 34], as well as corneal pigmentation changes [35]. However, our patient had not taken this medication.

As the rate of breast cancer diagnosis increases, the number of patients taking these medications continues to rise. With AIs in particular, there are often issues with adherence to therapy due to side effects [36], and at the same time, higher rates of adverse effects have been associated with lower risk of breast cancer recurrence [37]. It is important that we continue to investigate the effects of these agents and the resulting hormonal changes on various tissues in the body in order to better detect and manage any possible adverse effects.
